# Risk for arterial thrombosis after liver transplantation with hepatic artery reconstruction

**DOI:** 10.1093/bjsopen/zrab146

**Published:** 2022-01-31

**Authors:** Mohamed Ghazaly, Pulkit Sethi, Manikandan Kathirvel, Navneet A. Tiwari, Manoj Thillai, Rohit Gaurav, Veena Surendrakumar, John O. O. Ayorinde, Michael Allison, Sara Upponi, Christopher J. Watson, Raaj K. Praseedom, Paul Gibbs, Kourosh Saeb-Parsy

**Affiliations:** 1 Transplant Unit, Addenbrooke’s Hospital, Cambridge, UK; 2 Department of Surgery, Tanta University, Tanta, Gharbia, Egypt; 3 Department of Hepatology, Cambridge Biomedical Research Centre, Addenbrooke’s Hospital, Cambridge, UK; 4 Department of Radiology, Addenbrooke’s Hospital, Cambridge, UK; 5 Department of Surgery, University of Cambridge, and Cambridge NIHR Biomedical Centre, Cambridge, UK

*Dear Editor*

Knowledge of anatomical variations is important during the procurement of deceased donor organs to avoid organ damage and to promote complex vascular reconstruction^1^. Reconstruction of the hepatic artery is hindered by increased number of anatomical variants of the donor hepatic artery that could exist in up to 50 per cent of liver grafts^2^. The liver’s arterial supply is very complex and each one of the eight segmental arteries can possibly derive separately from the aorta^3^. Vascular complications following liver transplantation exacerbate postoperative morbidity^4,5^. The aim of this study was to assess the impact of hepatic artery reconstruction (HAR) on hepatic artery thrombosis (HAT) after liver transplantation (LT) and subsequent recipient morbidity and mortality rates.

Some 244 LTs were performed at Addenbrooke’s Hospital, Cambridge, UK between 2014 and 2017 with a median follow-up of 30 months (range 12–48 months). HAT occurring following LT was ascertained with CT. Donor and recipient variables were outlined, and outcomes were compared between recipients with and without HAR (*Supplementary material, Appendix S1*).

In the case of donor aberrant arterial anatomy of the graft (*Fig. S1*), back-bench reconstruction was carried out (*Fig. S2*). All patients were discharged on aspirin 75 mg once daily orally long-term. Only patients with vascular reconstruction who were considered at higher risk of graft vessel thrombosis received long-term formal anticoagulation (initially consisting of therapeutic dose dalteparin, followed by oral anticoagulation).

Liver transplants with and without HAR were largely comparable in terms of donor and recipient characteristics (*Table S1*). Within the surgical/operative parameters, operative time, arterial anatomy, and Roux loop biliary reconstruction were all more frequent in the HAR group (*P* = 0.007, 0.001, and 0.008 respectively) (*Table S2*).

Aberrant donor arterial anatomy was found in 76 donors (30.8 per cent), where 32 (42 per cent) had accessory, replaced right hepatic artery, and 30 (40 per cent) had accessory replaced left hepatic artery. Forty-eight grafts out of these seventy-six required arterial reconstructions: 27 had coeliac artery (CA) anastomosed to superior mesenteric artery (SMA), eight had aberrant right hepatic artery (ARH) to gastroduodenal artery stump, five had ARH to splenic stump, three had aberrant left hepatic artery to splenic stump, two had ARH to CA, two had extension common iliac artery grafts, and one graft had common hepatic artery to SMA (*Fig. S2*).

Overall, 20 of 244 patients had HAT (8.2 per cent; 13 of which were early, occurring within 4 weeks and 7 were late). HAT was significantly more common in the HAR group (18.8 *versus* 5.6 per cent; *P* = 0.0076; *Table S3*), although HAR did not increase the incidence of morbidity or death (*Fig. S3*). Nine patients in the HAR group had HAT, and all six early HATs in the HAR group needed retransplantation with no 30-day deaths.

Univariable regression analysis showed HAR (*P* = 0.001), abnormal arterial anatomy (*P* = 0.024), operative time (*P* = 0.042) and ischaemic cholangiopathy (*P* = 0.002) as risk factors for HAT. Multivariable analysis revealed HAR (odds ratio 2.31 (95 per cent c.i. 1.16 to 4.33), *P* = 0.015) and ischaemic cholangiopathy (odds ratio 2.64 (95 per cent c.i. 1.99 to 6.93), *P* = 0.021) as risk factors for HAT (*Table 1*). There was no significant difference in patient survival (*Fig. S3*).

**Table 1:** Univariable and multivariable analysis of risk factors for HAT

Risk factors	Odds ratio	*P*
**Univariable analysis**		
HAR	3.88 (1.51, 10.00)	0.001
Recipient age	0.69 (0.30, 9.63)	0.351
Donor type	1.18 (0.46, 3.00)	0.681
Donor age	0.85 (0.30, 4.33)	0.448
Donor BMI	2.41 (0.39, 7.66)	0.299
HCC	0.84 (0.23, 3.00)	0.539
Graft steatosis	0.63 (0.37, 4.53)	0.497
Cold ischaemia time	1.39 (0.84, 3.62)	0.195
Warm ischaemia time	0.85 (0.27, 2.50)	0.237
Operative time	2.62 (1.60, 5.31)	0.042
Abnormal arterial anatomy	2.97 (1.18, 7.51)	0.024
Aortic conduit	3.03 (0.91, 10.08)	0.106
Type of biliary reconstruction	0.79 (0.29, 2.13)	0.367
PV conduit	1.25 (0.15, 10.41)	0.521
Bile leak	0.78 (0.20, 6.27)	0.415
Isolated biliary stricture	1.90 (0.51, 7.05)	0.317
Ischaemic cholangiopathy	4.86 (1.52, 15.61)	0.002
Re-exploration	1.05 (0.34, 3.31)	0.729
**Multivariable analysis**		
HAR	2.31 (1.16, 4.33)	0.015
Operative time	1.50 (0.06, 4.22)	0.163
Abnormal arterial anatomy	1.68 (0.86, 3.26)	0.094
Ischaemic cholangiopathy	2.64 (1.99, 6.93)	0.021

Values in parentheses are 95 per cent confidence intervals. HAR, hepatic artery reconstruction; HCC, hepatocellular carcinoma; PV, portal vein.

This study has some limitations including small size and probable confounding factors. Moreover, the authors did not analyse arterial resistive index on Doppler ultrasound. The need for bigger multicentre prospective studies is emphasized by these constraints. Nonetheless, the findings indicate that, although a higher risk of HAT is strongly related to bench arterial reconstruction for an aberrant donor arterial anatomy, patient survival can be comparable to that of the control group.

## Supplementary Material

zrab146_Supplementary_Data
