# Supplementary material for: Risk for arterial thrombosis after liver transplantation with hepatic artery reconstruction
Source: BJS Open. 2022 Jan 31;6(1):zrab146. doi: 10.1093/bjsopen/zrab146 (PMC8830758; doi:10.1093/bjsopen/zrab146)
Supplement: zrab146_Supplementary_Data [file zrab146_supplementary_data.zip › Supplementary_material.docx]

**Supplementary Material**

**Materials and methods**

Patient were identified by interrogating a prospectively maintained electronic database. Patients receiving re-transplants were excluded from the study. HAT occurring post-LT was identified as an absence of doppler ultrasound arterial flow in the main hepatic artery, initiated either by routine checks or lab abnormalities and ascertained by CT scan. Donor and recipient variables were outlined, and outcomes were compared between recipients with and without hepatic artery reconstructions.

Back-bench reconstruction was undertaken by senior surgeons when required (This was not formally measured but is estimated at 20-40 minutes). The vessel sizes ranged from 2-5 mm, although these were not formally measured. Reconstruction was carried out under 2.5-4.5 magnification using interrupted 6’0 or 7’0 prolene depending on the size of the vessels. A consecutive revascularization was performed, and the graft was perfused firstly through the portal vein, followed by anastomosis and perfusion through the hepatic artery (using interrupted or continuous 5’0 or 6’0 prolene depending on the size of the vessel). Biliary reconstruction was performed after a haemostatic pause in the form of duct to duct or roux loop when indicated. The standard immunosuppression protocol consisted of intra-venous methyl prednisolone at end of anhepatic phase, with prednisolone, azathioprine or mycophenolate mofetil, and tacrolimus from day one.

Doppler ultrasound was routinely used to check postoperative arterial patency; within the first 24 hours post-transplant with specific emphasis on visualisation of arterial inflow at the porta hepatis and in both lobes of the liver, repeated at the end of the first week. However specific features of the arterial flow on doppler ultrasound, such as resistive index, were not systematically measured. Additional examinations were carried out if any clinical/lab concerns. Outcomes were compared between recipients with and without hepatic artery reconstructions using 2-tailed t-tests and chi-squared tests on continuous and categorical data respectively. A p value less than 0.05 was considered as statistically significant. Post-transplant survival was compared using Kaplan-Meir curves and the statistical significance was assessed using log rank test. Univariate and multivariate analysis were conducted to identify significant risk factors for HAT. Statistical analyses were performed using SPSS statistical software (IBM®, SPSS® Statistics 20, Chicago, IL, USA).

The small size of the cohort with HAT, together with centre-to-centre variations such as recipient and organ selection criteria and surgical technique should be considered when assessing the applicability of the results to other transplant centers.

**Results**

11 (14.5%) donors had both accessory left & right hepatic arteries, 2 (2.6%) had completely replaced circulation (Replaced Right Hepatic artery, Replaced Left Hepatic artery), and one donor (1.3%) had replaced right hepatic artery and accessory left hepatic artery (**Figure S1**).

Nine of the twenty patients with hepatic artery thrombosis (HAT) required re-transplantation. Hepatic artery reconstruction (HAR) did not increase the incidence of early allograft dysfunction (EAD), primary non-function (PNF), ITU/hospital stay, re-operation, and bile leak) or mortality (both 30-day and 1 year) **(Figure S3)**. The rescue procedure for confirmed HAT was re-transplantation.

**Table S1:** Donor and recipient characteristics in hepatic artery reconstruction (HAR) and non-HAR groups

| **Characteristic** | **HAR (n=48)** | **non-HAR (n=196)** |
| --- | --- | --- |
| Recipient Age (mean±SD) | 54 (±12) | 53 (±12) |
| Recipient Sex (male/female) | 29/19 | 122/74 |
| Previous surgery | 13 (27%) | 47 (24%) |
| History of Diabetes | 36 (75%) | 60 (30.6%) |
| Pre-transplant inotropes | 12 (25%) | 1 |
| Pre-transplant Haemodialysis | 16 (33.3%) | 2 |
| Pretransplant Mechanical ventilation | 12 (25%) | 2 |
| Previous TACE | 2 (4.2%) | 11 (5.6%) |
| Indication for transplant  NAFLD  ArLD  PSC  HCC  VH  PBC  others | 10  9  7  7  6  4  5 | 44  39  32  35  17  23  6 |
| Hepatocellular carcinoma | 7 (14.6%) | 35 (17.9%) |
| Previous PVT | 1 | 11 |
| Partial PVT | 4 | 14 |
| Donor age (Years) | 46 (±15) | 50 (±15) |
| Donor BMI | 26 (±3.8) | 27(±5) |
| Donor Na+ (mmol/L) | 148 (±9) | 148 (±7.5) |
| Blood group (ABO) | A(16), B(4), AB(1), O(27) | A(96), B(11), AB(7), O(82) |
| DBD/DCD | 32 (66.7%)/16 (33.3%) | 123 (62.8%)/73 (37.2%) |
| Graft steatosis  none  mild  moderate  severe  NK | 30  11  0  1  6 | 123  49  13  1  10 |

Data are presented as mean±SD or numbers (percentage); NK – not known;

TACE – transarterial chemoembolization; NAFLD - Non-alcohol fatty liver disease; ArLD - Alcohol related Liver Disease; PSC - Primary sclerosing cholangitis; HCC - Hepatocellular carcinoma; VH - Viral hepatitis; PBC - Primary biliary cirrhosis; BMI - Body Mass Index; PVT – portal vein thrombosis; DBD – donation after brain death; DCD – donation after circulatory death.

**Table S2:** Comparison of operative and technical parameters in hepatic artery reconstruction (HAR) and non-HAR groups

| **Operative/Technical** **Variables** | **HAR (n=48)** | **Non-HAR (n=196)** | **P** |
| --- | --- | --- | --- |
| Blood loss (ml) | 1517 | 1442 | 0.43 |
| Operative time (minutes) | 472 (±91) | 432 (±83) | **0.0073** |
| Cold ischemia (minutes) | 572 (±158) | 530 (±183) | 0.10 |
| Anastomotic warm ischaemia (secondary) | 43 (±12) | 45 (±13) | 0.61 |
| Abnormal arterial anatomy | 46 (95.8%) | 30 (15.3%) | **0.0001** |
| Biliary reconstruction (Roux Loop) | 24 (50%) | 59 (30%) | **0.0079** |
| Portal venous conduit | 1 (2%) | 9 (4.6%) | 0.69 |
| Aorto-hepatic conduit | 4 (8.3%) | 17 (8.7%) | 0.94 |
| Cavocavostomy/Classical/Piggyback | 44/3/1 | 154/40/2 | 0.079 |
| Arterial anastomosis (Continuous/interrupted) | 18 (37.5%)/ 30 (62.5%) | 72 (36.7%)/ 123 (62.8%) | 0.99 |
| Split liver transplant | 4 (8.3%) | 10 (5.1%) | 0.64 |
| Machine perfusion | 7 (14.6%) | 30 (15.3%) | 0.99 |

Data are presented as mean±SD or numbers (percentage)

**Table S3:** Comparison of recipients’ outcomes in hepatic artery reconstruction (HAR) and non-HAR groups

| **Outcome** | **HAR (n=48)** | **Non-HAR (n=196)** | **p** |
| --- | --- | --- | --- |
| Hepatic artery thrombosis (n=20, **8.19%**)  Early (n=13, 5.32%)  Late (n=7, 2.86%) | 9 (18.8%)  6 (12.5%)  3 (6.3%) | 11 (5.6%)  7 (3.6%)  4 (2%) | **0.0076** |
| Bile leak | 3 (6.3%) | 12 (6.12%) | 0.99 |
| Isolated stricture | 7 (14.6%) | 15 (7.7%) | 0.2264 |
| Ischaemic cholangiopathy | 4 (8.3%) | 14 (7.1%) | 0.99 |
| Early Allograft Dysfunction | 7 (14.6%) | 36 (18.4%) | 0.6852 |
| Primary Non function | 1 (2.1%) | 4 (2%) | 0.99 |
| Acute rejection | 9 (18.8%) | 37 (18.9%) | 0.99 |
| Sepsis | 7 (14.6%) | 61 (31.1%) | 0.1566 |
| Re-exploration | 10 (20.8%) | 37 (18.9%) | 0.9174 |
| ICU stay (mean days) | 1 | 5 | 0.4945 |
| Hospital Stay (mean days) | 21 | 23 | 0.6653 |
| Re-transplantation | 6 (12.5%) | 13 (6.6%) | 0.29 |
| Mortality (30-day) (n=3, 1.23%) | 2 (4.2%) | 1 (0.5%) | 0.1816 |
| Mortality (1-year) (n= 12, 4.91%) | 2 (4.2%) | 10 (5.1%) | 0.1938 |

Data are presented as numbers (percentage)
