# Supplementary figures and images for: Risk for arterial thrombosis after liver transplantation with hepatic artery reconstruction
Source: BJS Open. 2022 Jan 31;6(1):zrab146. doi: 10.1093/bjsopen/zrab146 (PMC8830758; doi:10.1093/bjsopen/zrab146)

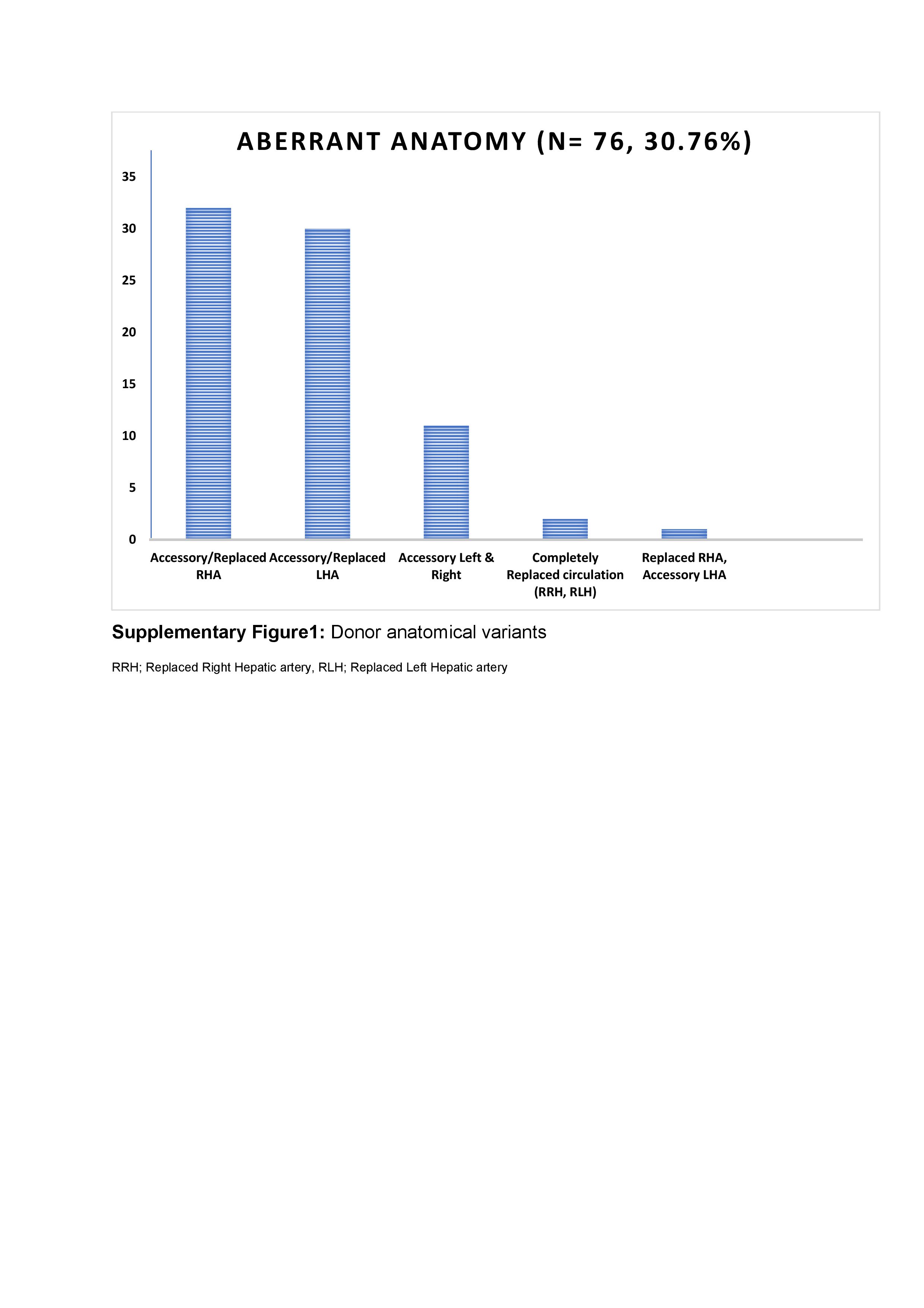

Supplement: zrab146_Supplementary_Data [file zrab146_supplementary_data.zip › Supplementary_Figure_1.jpg]

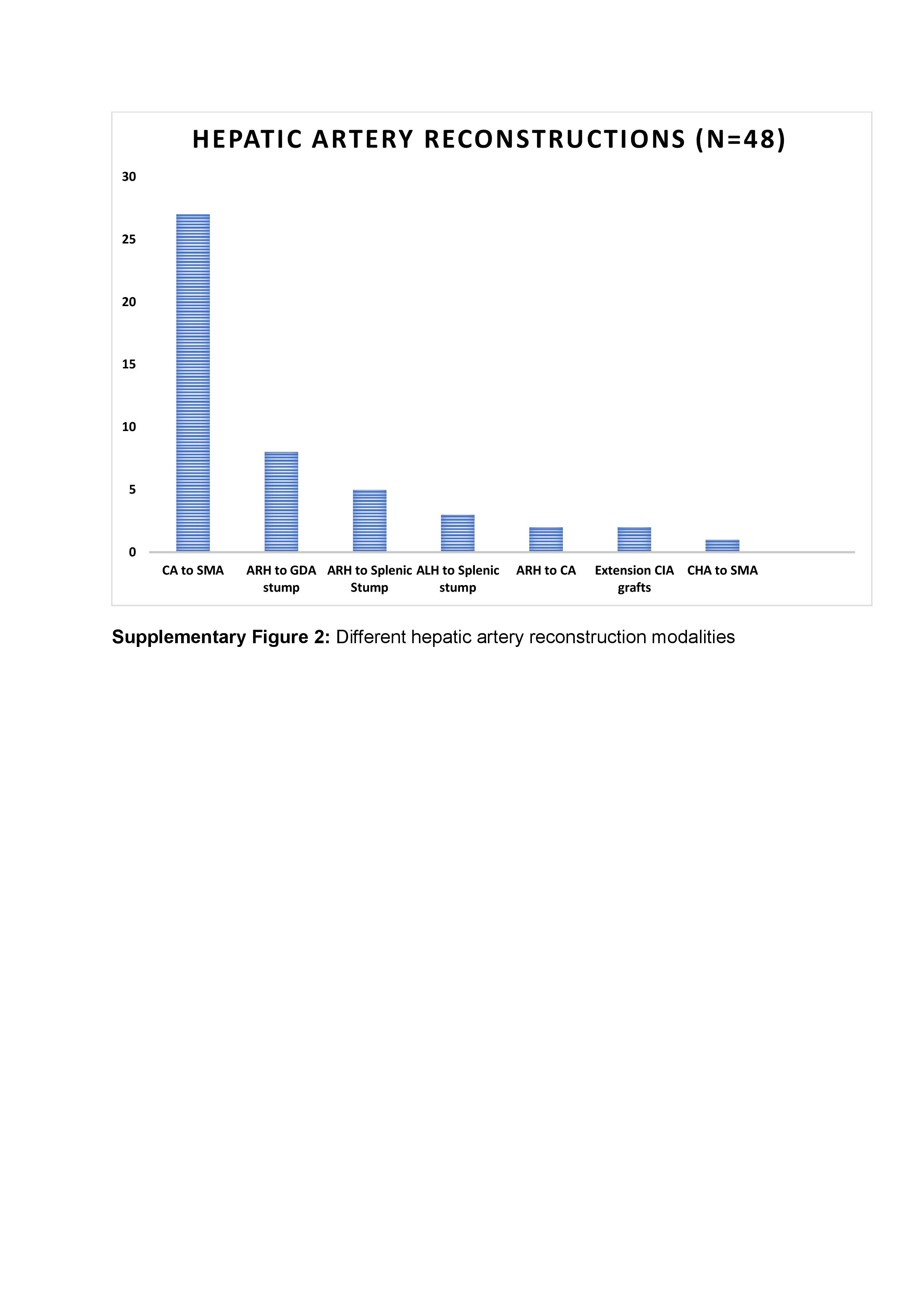

Supplement: zrab146_Supplementary_Data [file zrab146_supplementary_data.zip › Supplementary_Figure_2.jpg]

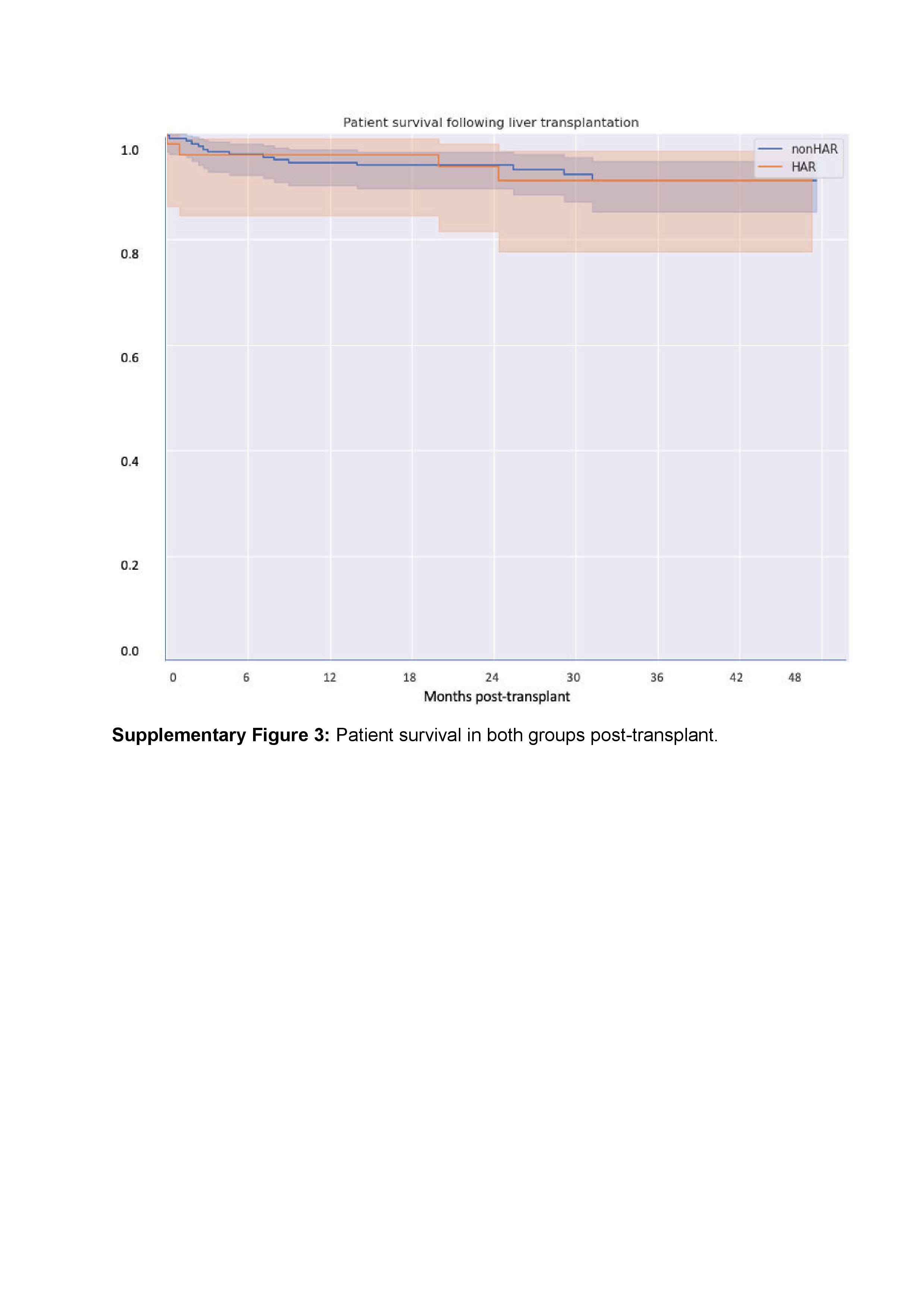

Supplement: zrab146_Supplementary_Data [file zrab146_supplementary_data.zip › Supplementary_Figure_3.jpg]
